# Mold sensitization is common amongst patients with severe asthma requiring multiple hospital admissions

**DOI:** 10.1186/1471-2466-5-4

**Published:** 2005-02-18

**Authors:** B Ronan O'Driscoll, Linda C Hopkinson, David W Denning

**Affiliations:** 1 Department of Respiratory Medicine, Salford Royal Hospitals NHS Trust, Hope Hospital, Salford M6 8HD, UK; 2 Department of Medicine University of Manchester Clinical Sciences Building Hope Hospital Salford M6 8HD UK

## Abstract

**Background:**

Multiple studies have linked fungal exposure to asthma, but the link to severe asthma is controversial. We studied the relationship between asthma severity and immediate type hypersensitivity to mold (fungal) and non-mold allergens in 181 asthmatic subjects.

**Methods:**

We recruited asthma patients aged 16 to 60 years at a University hospital and a nearby General Practice. Patients were categorized according to the lifetime number of hospital admissions for asthma (82 never admitted, 53 one admission, 46 multiple admissions). All subjects had allergy skin prick tests performed for 5 mold allergens (*Aspergillus, Alternaria, Cladosporium, Penicillium *and *Candida*) and 4 other common inhalant allergens (*D. pteronyssinus*, Grass Pollen, Cat and Dog).

**Results:**

Skin reactivity to all allergens was commonest in the group with multiple admissions. This trend was strongest for mold allergens and dog allergen and weakest for *D. pteronyssinus*. 76% of patients with multiple admissions had at least one positive mold skin test compared with 16%-19% of other asthma patients; (Chi squared p < 0.0001). Multiple mold reactions were also much commoner in the group with multiple admissions (50% V 5% and 6%; p < 0.0001). The number of asthma admissions was related to the number and size of positive mold skin allergy tests (Spearman Correlation Coefficient r = 0.60, p < 0.0001) and less strongly correlated to the number and size of non-mold allergy tests (r = 0.34, p = 0.0005). Hospital admissions for asthma patients aged 16–40 were commonest during the mold spore season (July to October) whereas admissions of patients aged above 40 peaked in November-February (Chi Squared, p < 0.02).

**Conclusion:**

These findings support previous suggestions that mold sensitization may be associated with severe asthma attacks requiring hospital admission.

## Background

Most asthma patients have mild symptoms which are well controlled with anti-inflammatory and bronchodilator therapy but a minority of asthma patients have severe airway inflammation and airflow obstruction requiring multiple hospital admissions. The reasons for these differences in asthma severity are complex and not fully understood [1]. At least two thirds of asthmatic patients are atopic with skin reactivity to common allergens [2-4]. It has also been reported that individuals with severe ("brittle") asthma may have a greater degree of atopy than other asthmatic patients [5]. Skin reactivity to fungal allergens such as *Alternaria *species has been reported to be especially common in patients with life-threatening asthma [6]. Asthma deaths, hospital admissions, respiratory symptoms, and Peak Expiratory Flow rates can be adversely affected by high fungal spore concentrations in outdoor air [7-11]. Mold sensitivity has been associated with increased asthma severity and intensive care admissions in adults and with increased bronchial reactivity in children [12-17].

Indoor mold exposure might also contribute to asthma severity. Many patients believe that their asthma is aggravated by damp housing, especially if there is visible mold growth [18]. It has been reported that asthma patients are more likely than control patients to live in a damp dwelling and their asthma severity was correlated to the degree of dampness and mold growth in their home (measured by a building surveyor) [19]. It is also known that asthma deaths in young adults in England and Wales are commonest in the months of July, August and September which coincides with peak levels of mold spores in the outdoor air in the UK [20-22].

These studies prompted us to undertake a study of asthmatic patients in Salford in Greater Manchester (North-West England) to assess whether atopy in general and mold sensitization in particular was associated with increased asthma severity. There are few agreed definitions of severe asthma so we used hospital admissions and treatment category (according to British Thoracic Society Asthma Guidelines) as surrogate markers for asthma severity [23].

## Methods

This study was undertaken in Hope Hospital, a 900 bedded University Hospital in Salford, Greater Manchester, UK. The study was designed to record the atopic status for mold and non-mold allergens of asthma patients at every level of severity from very mild to very severe (defined as multiple hospital admissions despite intensive asthma medication). We studied patients with severe asthma during hospital admissions or at subsequent visits to the hospital chest clinic. Patients were recruited opportunistically during hospital admissions or during routine consultations with the Respiratory Nurse Specialist (RNS) in the hospital Chest Clinic. Recruitment was evenly spread over 30 months from January 1996 to June 1998. During this period, the Respiratory Nurse service was under development with only one part-time RNS available to serve a large population of hospital in-patients and ambulatory care patients at chest clinics. Ward-based doctors and nurses referred patients with acute asthma to the RNS on an opportunistic basis based on availability of the part-time RNS and whether the ward teams were aware of the developing RNS service. About 20% of all adult asthma patients admitted during the study period were referred to the RNS and most of these were recruited in the study (provided the RNS had time available to do so). Although recruitment was not systematic (based mainly on availability of RNS service), we were not aware of any potential bias which might have affected the recruitment process and we believe that the recruited patients were typical of adult patients with acute asthma admitted to our hospital. We recruited ambulatory patients (some of whom had previous hospital admissions) at the same hospital chest clinic and in a single Primary Care practice where 25 patients were recruited during routine consultations with the practice nurse. This recruitment was also opportunistic, based mainly on the availability of time to complete the study protocol during busy clinics. We believe that these patients were typical of patients seen at hospital chest clinics and General Practice asthma clinics in this area. Patients were grouped according to their lifetime history of hospital admissions for asthma (multiple admissions, single admission, or no admissions). Patients with no hospital admissions were further categorized according to the treatment steps described in the British Thoracic Society guidelines for asthma management; Step 1 requiring only occasional bronchodilator treatment, Step 2 requiring low-doses (<800 mcg per day) of inhaled steroid, Step 3 requiring high doses of inhaled steroids or long acting beta agonists, Step 4 requiring additional therapy such as domiciliary nebulized therapy [23]. We attempted to recruit approximately equal numbers of patients at BTS steps 1, 2 and 3–4 to allow analysis of mold sensitization according to severity in non-admitted patients as well as in admitted patients.

All patients were Caucasians who were lifelong residents of the United Kingdom. Inclusion criteria were a diagnosis of asthma by the patient's doctor, age 16 to 60 and ability to give informed consent. All subjects gave written informed consent prior to partaking in the study which was approved by the Salford Research Ethics Committee. Exclusion criteria included a diagnosis of COPD, non-European ethnic group (98% of Salford residents are Caucasian) and consumption of any antihistamine in the previous 48 hours. All subjects completed a questionnaire concerning respiratory symptoms, smoking status and allergies.

All tests were conducted by one author (LCH) or by one other respiratory nurse specialist using a standardized technique in an open manner. A small drop of allergen was placed on the volar surface of the forearm. Allergens were purchased from Allergopharma (Reinbek, West Germany), a single batch of each allergen was used throughout the study. We used skin test lancets with a 1 mm tip (Bayer Prick Lancetter supplied by Miles Pharmaceutical Division, Spokane, Washington, USA). The lancet was introduced vertically into the skin through the allergen solution. Allergens studied were: negative control, histamine 0.1%, *Dermatophagoides pteronyssinus*, cat, dog, mixed grass pollen, *Aspergillus fumigatus*, *Alternaria alternata*, *Cladosporium herbarum*, *Penicillium notatum *and *Candida albicans*.

Weal diameter (if any) was recorded at 15 minutes. If a weal was asymmetrical, the mean of two perpendicular measurements was calculated. Weals less than 3 mm greater than the negative control reaction were regarded as negative in accordance with guidance issued by the European Academy of Allergology [24]. We devised an arbitrary numerical "sensitization score" for mold sensitization and non-mold sensitization to compare the number and size of positive allergy tests between groups of patients. This "sensitization score" was the sum of all positive weal diameters for each individual patient after subtraction of the negative control. For example, a patient with a 1 mm reaction to negative control, a 6 mm reaction to *Aspergillus *and a 5 mm reaction to *Cladosporium *would have a "mold sensitization score" of (6-1)+(5-1) = 9 mm.

As a subsidiary study (not part of the initial trial protocol), we also studied the seasonality of asthma admissions in Salford by reviewing the electronic records of 520 asthma admissions under the care of two pulmonary physicians who kept a computerized database at this hospital between 1995 and June 2000 (approximately 30% of all adult asthma admissions to the hospital). This covered a wide time-span before, during and after the period of the mold sensitization study; some of the allergy study patients were admitted and recruited during this time but they represented only a small (and random) proportion of the admissions studied for seasonality. Asthma admissions were analyzed by month of admission and divided into 3 four-month "seasons"; March to June (Spring and early summer season with maximum airborne levels of shrub, tree and grass pollens; July to October (late summer and fall season with maximum airborne levels of mold spores); November to February (winter peak of general respiratory infections involving COPD and older asthma patients); [20-22]. Patients were analyzed in two age groups as it is known that asthma admissions and asthma deaths in Britain are commonest in July to September for patients aged under 35 but older patients are more likely to suffer asthma death in winter [20,21]. We used two age bands (16–40 and >40) because our patients aged 35–40 had a seasonal profile for asthma admissions which was identical to the 16–35 group and different to the group aged above 40.

Statistical analysis was performed using Prism II software (GraphPad Prism, San Diego, California, USA). Chi squared test was used to compare the number of positive allergy skin tests between groups of asthma patients. Spearman Correlation Coefficient was used to compare each patients lifetime number of hospital admissions with their "Mold sensitization score" (as described above). Mann Whitney tests were used to compare mean sensitization scores between groups of patients.

## Results

One hundred eighty-one asthmatic patients were recruited. Their characteristics are given in table 1. No subject was on antihistamine medication and none had a negative reaction to histamine. No subjects had dermatographism (>3 mm reaction to negative control skin test). No eligible patient declined to partake in the study. There was a predominance of female subjects in all groups. Patients with hospital admissions were more likely to report current smoking than patients with no admissions (p = 0.03). The habit was commoner amongst patients with one admission than those with multiple admissions but this difference was not significant (p = 0.18). Patients with multiple admissions were more likely to have developed their asthma in childhood and they had a stronger family history of asthma.

**Table 1:** Patient characteristics.

	**Asthma No Hospital Admissions**	**Asthma One Hospital Admission**	**Asthma >1 Hospital Admission**
**Number**	82	53	46
	*32 BTS step 1 (Ref 23)*		
	*25 BTS step 2*		
	*25 BTS steps 3–4*		
**Percent male**	35%	25%	46%
**Mean Age (Range)**	37	36	36
	16–59	17–58	16–60
**%Smokers**	15%	34%	22%
**%Ex-Smokers**	29%	23%	17%
**%Non-Smokers**	56%	43%	61%
**Asthma onset before age 16 (Percent)**	44%	23%	70%
**Family history of Asthma in parents, siblings or children (%)**	56%	55%	76%

**Figure 1:**
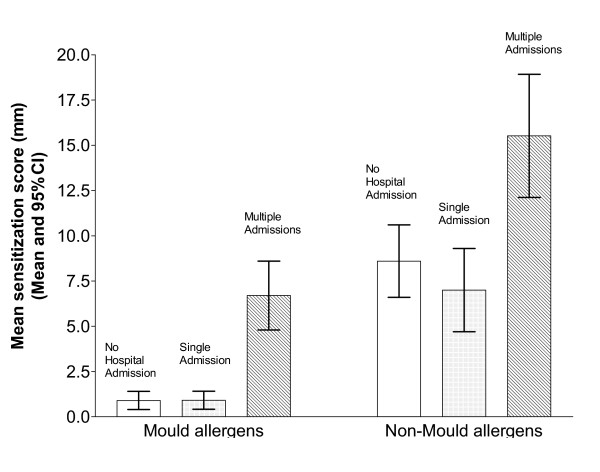
Mean mold sensitization scores and mean non-mold sensitization scores for asthma patients and controls (mean and 95% CI) Clear bar: 82 asthma patients with no hospital admissions. Grey bar : 53 patients with one hospital admission Striped bar: 46 patients with more than one hospital admission.

Positive skin tests to all allergens were commoner in the group with severe asthma (multiple hospital admissions) than patients with milder asthma. (Table 2 and Figure 1). Atopic sensitization was common in all groups, especially the severe asthma group This tendency was most marked for dog sensitization (Table 2). Dog ownership was 31% amongst patients with mild asthma (no admissions) and 30% amongst those with multiple admissions (who were more dog-allergic).

**Table 2:** Prevalence of mold and non-mold sensitization in asthma patients. *"Sensitization score" refers to number and size of positive skin tests as defined in methods section*.

	**Asthma No admissions**	**Asthma One Admission**	**Asthma >1 admissions**	**Chi squared p value**
**Mold allergens**				
***Aspergillus***	7%	6%	37%	<0.0001
***Alternaria***	5%	6%	26%	<0.0001
***Cladosporium***	1%	0%	41%	<0.0001
***Penicillium***	2%	4%	30%	<0.0001
***Candida***	10%	9%	33%	0.001
**Any mold sensitization**	16%	19%	76%	<0.0001
**>1 mold sensitisation**	5%	6%	50%	<0.0001
**Mean mold sensitization score (95% CI)**	0.9 mm 0.4–1.4	0.9 mm 0.3–1.5	6.7 mm 4.8–8.5	Mann Whitney See below
**Other allergens**				
***D pteronyssinus***	56%	47%	67%	0.13
**Grass pollen**	46%	38%	63%	0.025
**Cat**	37%	36%	59%	0.029
**Dog**	18%	19%	48%	0.005
**Any non-mold Sensitisation**	70%	47%	74%	0.008
**>1 non-mold sensitisation**	43%	38%	70%	0.002
**Mean non-mold sensitization score (95% CI)**	8.6 mm 6.6–10.6	7.0 mm 4.7–9.3	14.5 mm 11.1–17.9	Mann Whitney See below

Mann Whitney Analysis of "Sensitization Scores" between different categories of asthmatic patients.

Mold sensitization score:

No admission V single admission P = 0.87

No admission V multiple admissions P = < 0.0001

Single admission V multiple admissions P = < 0.0001

Non- Mold sensitization score:

No admission V single admission P = 0.29

No admission V multiple admissions P = < 0.009

Single admission V multiple admissions P = 0.002

Mold sensitization was uncommon in mild asthma but very common in asthma patients with multiple admissions (Figure 1 and Table 2). There was a striking difference in the prevalence of mold sensitization amongst the three asthma groups. Three quarters of patients with multiple hospital admissions were sensitized to molds and half of them reacted to multiple mold allergens. The patients with a single hospital admission were more similar to those with no admissions than to the multiple admission group. This trend was seen for all five mold allergens studied (table 2). The frequency of sensitization to any individual mold ranged from 26% (*Alternaria*) to 41% (*Cladosporium*) in the severe asthma group compared with 0–10% in the milder asthma groups.

*Aspergillus *and *Candida *precipitins and specific IgE were not measured in this study. None of these patients had any clinical features suggestive of allergic bronchopulmonary aspergillosis (ABPA) such as pulmonary infiltrates, bronchiectasis or marked eosinophilia. The number of admissions correlated with the number and size of positive skin tests using the scoring system described previously. For mold sensitization, the Spearman Correlation Coefficient was 0.60 (two-tailed p < 0.0001) and for non-mold allergens was 0.34 (two-tailed p = 0.0005). The cumulative "mold sensitization score" and the "non-mold sensitization score" for each group of patients is shown in table 2. Only two of the 99 patients with asthma admissions had ever required admission to an Intensive Care Unit. Both were sensitized to a single mold (one *Aspergillus*, one *Penicillium*).

Of the patients not admitted to hospital, 32 had very mild asthma (BTS Step 1), 25 had mild-moderate asthma (BTS Step 2) and 25 had moderate to severe asthma (BTS Steps 3–4). There was no significant difference in mold or non-mold sensitization between these groups of non-admitted patients with different grades of asthma severity.

**Figure 2:**
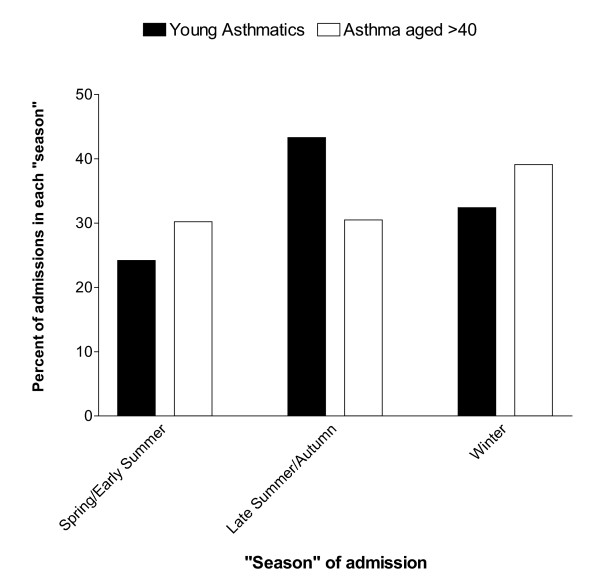
Asthma admissions aggregated by "season", comparing 16–40 age group (black bars) with age >40 (white bars).

Our review of asthma admissions to this hospital between 1995 and 2000 identified 520 patients admitted under the care of the two chest physicians who kept a computerized database (approximately 30% of all asthma admissions to the hospital). There were 173 asthma admissions in the 16–40 age group, these admissions peaked in late summer and fall (figure 2). Of these admissions 24.3% occurred between March and June, 43.4% between July and October and 32.4% between November and February. By contrast, 347 asthma patients aged over 40 had a winter peak of admissions (30.3% March-June, 30.5% July-Oct, 39.2% Nov-Feb) These patterns of admissions were significantly different (Chi squared p < 0.02). The summer-fall peak in the 16–40 age group amounted to 33 additional admissions above the spring baseline. This represented 6.4% of all asthma admissions or 19.1% of admissions in the 16–40 age group.

## Discussion

Although the present study was larger than most previous studies of mold sensitization in severe asthma, it must be regarded as a "pilot study" due to the non-systematic recruitment of asthma patients and the cross-sectional nature of the study. Our data indicate that mold (and dog) sensitization is common in patients with severe asthma requiring multiple hospital admissions in Manchester. The results are consistent with previous evidence that atopy (especially to mold allergens) is related to asthma severity or bronchial hyper-reactivity [4-6,12-17]. A recent cross-sectional study of 1132 adults with asthma found that sensitization to *Alternaria *or *Cladosporium *is a powerful risk factor for severe asthma[16] in several European countries and also in Australia, New Zealand and in Portland, Oregon. The link between dog sensitization and asthma severity is in agreement with previous studies [5,14]. We had also expected to find an excess of house dust mite (*D. pteronyssinus*) sensitization in our patients with more severe asthma [15,25]. However, reactivity to this allergen was common in all asthma groups and only slightly commoner in patients with multiple admissions.

There has been some debate about the best cut-off point for weal size to define a positive skin-prick test. We accepted the European Academy of Allergology figure of 3 mm greater than the negative control [24]. However, re-analysis of our data using a 2 mm or 4 mm difference from the negative control would make no difference to the results. Furthermore, the number and size of positive skin tests to mold allergens was greatest in patients with a high number of admissions suggesting that the relationship is a genuine one. It was also notable that, although sensitization to non-mold allergens was common in the informal control population and in patients with mild asthma, mold sensitization was uncommon in these groups. This indicates that the positive skin tests to mold allergens are unlikely to be due to irritant reactions of a non-allergic nature. As skin test reagents from different manufactures are not standardized, different results might be obtained with different manufacturers' reagents. Until such antigens are standardized, this remains unsatisfactory. However, the consistency between the present study and the recent European Community respiratory health survey (using different antigens) supports the validity of the association between mold sensitization and severe asthma.

A key question is whether severe asthma is actually caused by sensitivity to molds or is simply associated with it. In any case, mold sensitivity will certainly not be the only cause of severe attacks of asthma; upper respiratory tract virus infections and some drugs being two of other well documented causes. The greater degree of mold sensitization in the severe asthma group could simply reflect an extreme example of the generalized increase in atopy amongst this group. However, we believe that mold allergy may be responsible for severe asthma attacks for several reasons.

First, the temporal relationship between high environmental spore counts and asthmatic attacks is strong. Airborne spore levels may be up to 1000 times higher than pollen levels [26]. The data of Targonski and colleagues provide strong evidence that asthma deaths in Chicago are more likely to occur on days when local mold spore counts are high [7]. High mold spore counts have been associated with asthma admissions in New Orleans (adults) and in Derby, UK (adults and children) [10,11]. Asthma symptoms are increased in California and Pennsylvania on days when mold spore counts are high [8,9]. The young patients of O'Hollaren and colleagues who were *Alternaria*-sensitive had their near-fatal asthma episodes in summer and early fall when mold spore levels would be expected to be high [6].

Second, the seasonal (summer -fall) peak of asthma admissions occurs when ambient air counts of molds are high. We have documented a late summer-fall peak of asthma admissions involving young adults in Manchester which coincides with the summer-fall peak of asthma deaths in UK patients aged under 35 years [20,21]. These asthma admissions also coincide with the peak months for outdoor levels of fungal spores [22,27]. Although there is no aero-biology service in Manchester, data for surrounding towns have shown a consistent summer-fall peak in mold spore counts. In Cardiff, for example, a city 150 miles south-west of Manchester with a similar climate, the highest spore counts were measured in late summer and fall [22]. The Cardiff authors reported maximal levels of *Cladosporium *in July, *Alternaria *and hyaline basidiospores in August, uredospores in September and coloured basidiospores in October. The data from Derby (52 miles south-east of Manchester) are similar[11]. In addition, similar findings have been reported from Copenhagen (600 miles north-east of Manchester) where 87% of the microfungal flora in outdoor air is accounted for by *Cladosporium*, *Alternaria*, *Penicillium *and *Aspergillus *with maximal levels between June and October [27]. A pan-European study with centres in Oregon, USA, Australia and New Zealand involving questionnaires and skin prick tests in 17,000 patients identified 1132 patients with asthma the severity of which could be determined [16]. Sensitisation to *A. alternata *and *C. herbarium *was common and associated with asthma severity – OR of 2.03 for the former, of 3.2 for the latter fungus and 2.34 for both. No such association was found for pollens or cats, although sensitization to house dust mite was slightly more frequent in those with severe asthma (OR 1.61) [16]. The present study extends these findings to a wider range of fungal allergens and a greater degree of asthma severity.

Third, there is evidence that indoor mold exposure may contribute to asthma severity. Many patients report respiratory symptoms in damp and moldy houses and a review of nine population-based studies found that seven reported one or more positive associations between fungal levels and health outcomes [28]. The study of Williamson and colleagues in Scotland reported that asthmatic patients were more than twice as likely than control patients to live in a house that was considered damp or moldy by a building surveyor [19]. Furthermore, in that study, there was a positive association between a patient's asthma severity and the degree of dampness which the surveyor measured in the patient's home (r = 0.3, p = 0.006) and independently with an index of visible mold growth in their dwelling (r = 0.23, p = 0.035). In a study of German children, it was found that bronchial hyper-reactivity was associated with damp housing [29] that was only partly explained by exposure to house dust mite antigen, suggesting that other factors such as mold growth may also be important. Taskinen et al found that the prevalence of asthma was similar (4.8%) amongst children attending a school with moisture and mold problems compared with a control school but asthma symptoms such as wheeze and cough were commoner in the damp moldy school as were emergency visits to hospital (OR 2.0, p < 0.01) [30].

Fourth, we know that *Aspergillus *in particular is a major respiratory allergen causing the vast majority of cases of allergic bronchopulmonary mycosis [31-33]. It is likely that these cases represent the extreme of a spectrum of mold allergy, the slightly less severe manifestation of which is severe asthma as described in this study and without all the serological and radiological markers characterisitic of ABPA. The reactivity of asthmatic patients to multiple mold allergens could be due to genuine sensitization to a variety of molds or it could be due to cross-reactivity between mold allergens. The paper of Hemmann and colleagues suggests that *Aspergillus *and *Candida *allergens may share IgE-binding epitopes [34]. However, it is believed that multiple mold sensitization skin test reactions are usually due to sensitivity to multiple antigens rather than cross reactivity [25]. Few fungi of the >1 million species of fungi thought to exist worldwide have been subjected to the antigenic scrutiny that *Aspergillus *and a few other common airborne fungi have and it is likely that sensitization to other fungi will be discovered in the future.

It is not known why mold allergens should produce more severe airway disease than other common allergens such as house dust mites, cat dander, or grass pollen. Fungi are very common in the environment and *Candida *is present in the gut of most, if not all, humans. The difference may relate to the nature or intensity of exposure to mold allergens or to their ability to become airborne and to gain entry to human airways due to their small size. Also many potent allergenic proteins have been described in *Aspergillus *and some in other fungi [35]. There is the probability that some fungal antigens, such as Asp f6 being a manganese dependant superoxide dismutase which is closely related to the human enzyme, might set up a self perpetuating allergic response, which is aggravated every time *Aspergillus *is inhaled, which is almost hourly. Some fungal antigens are proteases (Asp f5, Asp f10, Asp f13, Asp f15, Asp f18) and as DP1 is also a protease – a similar pathogenic role can be postulated [35,36].

It therefore seems likely that there is a causal relationship between mold allergy and asthma severity for some younger asthma patients. Our seasonal admissions data suggest that up to 6% of adult asthma admissions in Manchester and 19% of asthma admissions in the 16–40 age group may be attributable to mold allergy. These unlucky individuals seem to have more severe asthma than patients with sensitization to other common allergens and an increased risk of fatal or near-fatal asthma or hospital admissions during the mold spore season, especially on days when the local mold spore count is high. The risk may be further increased if the patient lives in a damp, moldy house or attends a damp moldy school [19,29,30,37]. It is not yet known which mold species are most important in causing such reactions or whether indoor or outdoor mold exposure is more important {outdoor levels are usually higher}[38]. It is very difficult to make accurate measurements of indoor mold exposure and most studies have used surrogate markers such as dampness or visible mold growth. This phenomenon needs to be studied further in a variety of geographic locations with different climates. It is not yet known if environmental modification or the use of airway protection would be of any value in the management of mold-allergic asthma patients.

One of the fungi which we investigated and for which antigenic extracts are available, *Candida albicans*, is a yeast, does not become airborne but does produce hyphae in tissue. Thus it is possible that human asthma due to fungal allergens may have three sources of allergen exposure – outdoor mold spores, indoor mold spores and endogenous fungal growth on body surfaces including the skin and gut [33].

Fungal sensitization is relatively uncommon in our British asthma patients and in Finnish schoolchildren [30] compared with Arizona [12] and Australia where up to 31% of asthmatic children and up to 23% of non-asthmatic controls react to at least one fungal allergen [15,17,37]. The prevalence of *Alternaria *sensitization in Italian patients with respiratory symptoms ranges from 2% in Northern Italy to 29% in Southern Italy [39]. Some of these differences are probably due to difficulties in the standardization of mold allergen extracts or skin testing techniques [26]. Also fungal sensitization is commoner in children and declines with age. However, it is likely that the significance of a positive skin test to fungal allergens varies in different climatic zones. Our data and those of O'Hollaren, Sureik and Black [6,16,17] suggests that fungal skin sensitization tests may identify adults who are at risk of especially severe asthma. The difference in the prevalence of mold sensitization between patients with multiple asthma admissions and the other groups in our study was so striking that it is extremely unlikely to have occurred by chance. Mold allergy tests may be useful to screen for children and adults who are at greatly increased risk of developing severe or fatal asthma. A large prospective study will be required to confirm these preliminary findings.

## Conclusion

The findings of this study support previous suggestions that mold sensitization may be associated with severe asthma attacks requiring hospital admission.

## Abbreviations

BTS Guidelines = British Thoracic Society Guidelines for Asthma Management, RNS = Respiratory Nurse Specialist

## Competing interests

The author(s) declare that they have no competing interests.

## Authors' contributions

ROD originated and co-ordinated the study and contributed to the analysis of the data and preparation of the paper.

LCH contributed to the design of the study and was the main clinical investigator. She also contributed to the analysis of the data and preparation of the paper.

DWD contributed to the design of the study and contributed to the analysis of the data and preparation of the paper.

## Pre-publication history

The pre-publication history for this paper can be accessed here:


